# Effects of Genotype on Pig Carcass, Meat Quality and Consumer Sensory Evaluation of Loins and Bellies

**DOI:** 10.3390/foods13050798

**Published:** 2024-03-05

**Authors:** Violeta Razmaitė, Rūta Šveistienė, Artūras Šiukščius

**Affiliations:** Department of Animal Breeding and Reproduction, Animal Science Institute, Lithuanian University of Health Sciences, R. Žebenkos 12, LT-82317 Baisogala, Lithuania; ruta.sveistiene@lsmu.lt (R.Š.); arturas.siukscius@lsmu.lt (A.Š.)

**Keywords:** pork, meat quality, consumer evaluation, pig genotype, carcass

## Abstract

The objective of the study was to compare carcass and meat quality among Lithuanian White, Lithuanian Indigenous Wattle and conventional hybrids as well as consumer sensory evaluations. The pigs were slaughtered at a live weight of approximately 95–100 kg. After 24 h of slaughter, carcasses were evaluated and sampled for the analysis of meat quality traits of loins and consumer evaluation of cooked loins and baked bellies. The pigs of both Lithuanian breeds had higher backfat thickness and depth of underbelly but lower loin area compared with hybrids. However, more fatty local breeds had higher intramuscular fat content but lower cholesterol content than hybrids. The loins from local pigs displayed higher pH and colour with lower lightness and yellowness but higher redness and also lower cooking loss, shear force and hardness than conventional hybrids. Pork from lean hybrids had a higher proportion of polyunsaturated fatty acids and more favourable lipid quality indices such as atherogenic (AI) and thrombogenic (TI) indices and the hypocholesterolemic/hypercholesterolemic (h/H) ratio; however, the peroxidisability index (PI) and iodine value (IV) were less favourable compared with local pigs. Consumers evaluated cooked loins and baked bellies. The loins from local breeds scored higher in juiciness, taste and overall acceptability compared with conventional hybrids. However, a higher overall acceptability was observed for the lean bellies of hybrids. The results can be used to increase pork consumption choices.

## 1. Introduction

Meat production and consumption are questioned due to the sustainability and ethical aspects of meat production and the health issues related to meat consumption [1]. Although it is predicted that pork consumption in Europe will be decreasing, even nine of eleven European countries currently report very high pork consumption [1]. The highest number of different pig breeds have also been developed, registered and exploited in Europe [2]. During the long breed formation period, the attitudes towards their phenotypes and production abilities have changed. Early artificial pig selection and improvement from the middle of the 18th century to the 20th century was associated with the creation of breeds with an increased penchant for fatness [3]. However, the 20th century was marked by an industrialisation process in agriculture, especially in its second half, and pig breeds were subjected to high selection pressures for lean production traits [4,5,6,7]. Concern for a healthier diet and rejection of animal fat in the diet of consumers [8] had a huge impact on local pig breeds, which have become out of use and were either largely replaced by highly selected international breeds or their crosses [4,6] and, as a result, many previously common breeds have become rare or even endangered [9]. Although commercial breeds are more efficient than local breeds, autochthonous breeds are, in general, well adapted to the production systems in which they have been constituted [6,10,11,12]. Moreover, pigs from local breeds have good quality [7,13,14,15,16,17,18] of meat used to produce both traditional and other various products. Local pigs are often produced in specific systems with outdoor or extensive conditions that contribute to the overall high acceptability of these systems and of their products by consumers [19,20,21,22] because, with the improvement of people’s living standards, meat consumption is gradually changing from quantity to quality and this implies the increased demand for specialised food of animal origin and requests a large variability of the species and breeds used. It is known that fatty Mangalitsa pigs are distinguished by their outstanding meat quality, which corresponds with current requirements and enjoys a renaissance owing to attempts to preserve traditional breeds [23].

The emphasis is on the use of indigenous pig breeds with the perspective of preserving pig genetic resources. As pig production is based on pigs that come from commercial crossbreeding programmes, comparative studies conducted in different countries with indigenous breeds and their hybrids with lean international breeds have shown higher fatness of local pigs and an opportunity to improve the carcass quality by crossing [6,10,23,24,25,26,27,28].

Despite the fact that the Lithuanian White pig breed was developed in the 20th century [29], its selection was aimed at breed adaptation for the production of both bacon and heavy fatty pigs [30,31]. Meanwhile, the artificial selection of Lithuanian Indigenous Wattle pigs was not carried out. Therefore, with the aim of finding ways to reduce the carcass fatness of Lithuanian pig breeds, studies [29,32,33,34] on crossbreeding, slaughter at a lower weight, and restricted feeding during the finishing phase were conducted. Although it is known that Lithuanian breeds showed lower performance [35] compared to conventional breeds, information is still lacking about meat quality comparisons between Lithuanian pig breeds and conventional hybrids. The purpose of this study was to compare the carcass and meat quality between Lithuanian White, Lithuanian Indigenous Wattle and conventional hybrid pigs and also to evaluate consumer sensory preferences.

## 2. Materials and Methods

### 2.1. Animals, Carcass Evaluation and Meat Sampling

The experiment was approved by the Board of the Animal Science Institute of the Lithuanian University of Health Sciences (protocol No. 23/01/30/01 of 30 January 2023).

The animals and their meat included in the present study were from the local pig breeds (16 Lithuanian Indigenous Wattle, 16 Lithuanian White and 16 conventional hybrids derived from crossings between Landrace and Yorkshire pig breeds). The pigs were offered commercial feed (AB Kauno Grūdai, Kaunas, Lithuania) consisting of wheat, barley, triticale, soy meal and sunflower meal (15.2% crude protein and 3.8% fat). The pigs were selected for experimental slaughterings at a live weight of approximately 95–100 kg on 2 different slaughter days, with 24 pigs on each day after 4 km transportation. Lairage lasted for approximately 2–2.5 h. The animals (24 females and 24 castrated males) were slaughtered according to standard procedures as described in our previous study [29].

Eviscerated carcasses without head, legs and tail were weighed to determine the warm carcass weight and chilled overnight at +2–4 °C. The dressing percentage was calculated as the percentage of carcass weight 30–45 min after slaughtering on live weight before slaughter. The carcasses were evaluated 24 h post mortem. The carcass length was measured in two ways: as the total length from the cranial edge of the first neck segment to the anterior edge of the *symphysis pubis* and as the bacon length from the cranial edge of the first rib to the anterior edge of the *symphysis pubis*. The measurements of midline backfat thickness were carried out with a ruler on the left side of the cold carcass at the crest, at 6/7, 10, at the last rib, at the thinnest lumbar backfat point and at three points over the *gluteus medius* (the anterior part, above the highest point and at the posterior part). The measurements of the ventral part of belly thickness were carried out along the line of teats at three points: 5 cm caudally from the last teat, parallel with the last thoracic vertebra and 3 cm caudally from the sternum and averaged. A picture was taken to record the shape of the *longissimus dorsi* (LD) at 1/2 lumbar vertebra, and loin and fat area were measured planimetrically as described in the previous study [29].

The left side of each chilled carcass was weighed and cut into three primal cuts (ham, shoulder and middle part). The hind leg was removed by cutting through the knee joint. The ham was separated by a straight cut at the next-to-last vertebrae, while the shoulder was separated between the 5/6 thoracic vertebrae. All these parts were weighed. Furthermore, loin and fat samples were taken for meat quality assessments from the 1/2 lumbar vertebra to the posterior part of LD. For consumer sensory analysis, the loin and belly were taken from the middle part of the carcass.

### 2.2. Meat Quality Assessments

#### 2.2.1. Functional Properties of LD Muscles

The ultimate pH of LD was determined 24 h after slaughter using a digital portable pH-meter PT-380 (Boeco, Hamburg, Germany) equipped with a glass electrode (Witeg Laboratory Technik GMBH, Wertheim, Germany) after calibration using pH 4.0 and 7.0 buffer solutions. Five colour parameters of LD in the CIE L* a* b* and L* C h colour spaces were measured using a chromameter CR-410 (Konica Minolta, Osaka, Japan). The measurements and specifications of the chromameter were described in our previous study [29].

The moisture loss of LD was measured in three ways: drip loss, thawing loss and cooking loss. The drip loss was assessed according to the EZ-drip loss method [36]. To determine the thawing and cooking losses, each LD sample was weighed before packaging, frozen, and stored in the freezer at −65 °C. After being frozen for four weeks, the samples were defrosted. After thawing at 4 °C for 24 h, the muscles were removed from the packaging, blotted with a paper towel and weighed. Then, the samples were cooked in thin-walled plastic bags at 80 °C for 1 h by immersion in a water bath with automatic temperature control [37], and then cooled at room temperature (20 ± 2 °C), weighed again and used for texture analysis. The thawing loss was calculated as a percentage difference between the initial pre-freezing weight and the post-thawing weight. The cooking loss is defined as the percentage of weight loss of the sample before and after cooking and cooling.

#### 2.2.2. Assessment of Chemical Composition

The chemical composition of LD (dry matter, crude protein, crude fat and ash contents) was determined according to methods no 950.46B, no 981.10, no 960.39 and no 920.153; AOAC, 1990 [38], which, as well as the used equipment specifications were described in our previous study [29]. The contents of protein, fat, and ash were expressed as the weight percentage of dry matter from muscle tissues.

The cholesterol content in LD muscle was determined according to the extraction method [39] and followed by a high-performance liquid chromatography (HPLC) separation and analysis on a Shimadzu 10 A HPLC system (Shimadzu Corp., Kyoto, Japan). The data collection and evaluation were performed by using an LC Solution (Shimadzu Corp., Kyoto, Japan) operating system. The analytical column was LiChrospher 100 RP-18e, 150 × 4.6 mm, 5 mm (Alltech Associates Inc., Chicago, IL, USA) with a guard column (LiChrospher 100 RP-18, 7.5 × 4.6 mm). The cholesterol content was expressed as mg/100 g fresh meat.

### 2.3. Instrumental Evaluation of Texture

The texture of LD was instrumentally measured by the Warner–Bratzler shear test (WBSF) and by the texture profile analysis (TPA) on the cooked samples using a Texture Analyser TA 1 (Measurement and Calibration Technologies Ametek Comp., Lloyd instruments, Largo, FL, USA) as described in the previous study [40].

### 2.4. Consumer Sensory Evaluation

The sensorial evaluation of pork loin and belly was performed two days after the carcasses maturation process with the participation of untrained panelists who declared themselves to be consumers of pork meat. Two repeated consumer panel sessions on different days with 45 consumers, including 25 females and 20 males were conducted in a large banquet room located in the basement of the mansion under artificial lighting. The consumers were mainly recruited from the staff members of the Animal Science Institute and from the community around the Baisogala area. The panel sessions lasted for about 1.5 h. Loin chops were cooked at 100 °C, and belly chops were baked in an electric oven (Universal temperature chamber SP-105, Kambic, Semic, Slovenia) at 180 °C until the core temperature reached 70 °C. Cooked and baked chops were cut into pieces of 1.5 cm and served on pre-heated plates, and each genotype sample was numbered. Each participant evaluated six samples (cooked loin and baked belly) of castrates related to 3 pig genotypes in blind conditions. The participants were also supplied with a ballot, fork and knife, cup, toothpick, napkins, water and palate cleansers (unsweetened tea and bread) to use between samples. Before the start of each panel, the participants were given verbal instructions regarding the ballot and usage of the palate cleansers. The participants were asked to score aroma, juiciness, flavour/taste and overall liking of the samples on a nine-pointing scale (1 point as dislike extremely and 9 points as like extremely).

### 2.5. Fatty Acid Profiles

The extraction of lipids for the fatty acid analysis was performed with a mixture of two volumes of chloroform (Chromasolv Plus for HPLC containing 0.5–1.0% ethanol as a stabiliser) and one volume of methanol [41]. Methylation of the samples was performed using sodium methoxide: 5 mL of 25 wt% solution in methanol was added to the sample and stirred. After 1 h, 7 mL HCL, 6 mL hexane and 2 mL H_2_O were added. The top layer was transferred into a new test tube and evaporated. Fatty acid methyl esters were prepared according to the procedure described in the literature [42]. The FAMEs were analysed using a gas–iquid chromatograph (GC—2010 SHIMADZU, Kyoto, Japan) fitted with a flame ionisation detector. The separation of methyl esters of fatty acids was affected on the capillary column Rt 2560 (100 m × 0.25 mm × 0.2 μm; Restek, Bellefonte, PA, USA) by temperature programming from 140 °C to 240 °C. The temperatures of the injector and detector were held, respectively, at 240 °C and 260 °C. The rate of flow of carrier gas (nitrogen) through the column was 0.79 mL/min. The peaks were identified by comparison with the retention times of the standard fatty acid methyl esters “37 Component FAME Mix” and trans FAME MIX k 110 (Supelco, Bellefonte, PA, USA). The relative proportion of each fatty acid was expressed as the relative percentage of the sum of the total fatty acids using “Lab solutions LC/GC” software, version 5.71, for Shimadzu gas chromatograph workstations.

The samples were analysed at least in duplicate for all analytes.

### 2.6. Lipid Quality Indices

Lipid quality indices, i.e., atherogenic index (AI) and thrombogenic index (TI), were calculated according to Ulbricht and Southgate [43]. The hypocholesterolemic/hypercholesterolemic (h/H) ratio was calculated according to Santos-Silva et al. [44]. The peroxidisability index (PI) was determined according to Du et al. [45].

Iodine value (IV) was calculated using the American Oil Chemists Society (AOCS) equation [46] as the sum of the concentrations of unsaturated fatty acids multiplied by their conversion factors [(C16:1 × 0.950) + (C18:1 × 0.860) + (C18:2 × 1.732) + (C18:3 × 2.616) + (C20:1 × 0.785) + (C22:1 × 0.723)].

### 2.7. Statistical Analysis

The carcass and meat quality data were subjected to the analysis of variance in the general linear (GLM) procedure in IBM SPSS Statistics for Windows, Version 29.01.0 (171), IBM Corp., Armonk, NY, USA with LSD tests to determine the significance of differences of means between the groups. The GLM univariate model included fixed factors of genotype group, gender and factor interactions (genotype × gender). The data of the consumer sensory evaluation were subjected to the analysis of variance in repeated measures of the general linear (GLM) procedure. The differences were regarded as significant when *p* < 0.05.

## 3. Results and Discussion

### 3.1. Characterisation of Slaughtered Pigs

Conventional hybrids with faster growth slaughtered at a similar live body weight were about 2.5 months younger (*p* < 0.001) compared with the pigs of both Lithuanian breeds (Table 1).

In our previous study [29], when pigs were treated with ad libitum and restricted feeding, the mean weight of LW at a similar age of 203.5 days was higher (108.6 kg); however, LIW pigs reached a similar weight of 98.7 kg at an older age (212.3 days) compared with the pigs used in the present study with standard feeding. Pig breeds and production of local pig breeds show a wide variability of their specific production systems [22,47], and only a small number of studies aimed at evaluating the breeds’ potential for growth and comparing their growth with the growth of commercial breeds. The results received in this study are consistent with the data of the comparison of commercial crossbred pigs with the pigs of the native British Large Black breed [48].

### 3.2. Carcass Evaluation

Despite a similar body weight at slaughter, conventional hybrids and LW pigs showed higher (*p* < 0.05) hot carcass weight and dressing percentage than the pigs of the LIW breed (Table 2). The lower carcass weight of the ancient LIW breed than that of LW and hybrid pigs corresponds with the findings of other authors who compared local Pulawska [24], Mangalitsa [23] and Cinta Senese [49] breeds with modern breeds. Conventional pigs had 6 and 3.3 cm higher (*p* < 0.001 and *p* < 0.01, respectively) total carcass length with neck compared with LIW and LW pigs. However, concerning the bacon length (carcass length without neck), there was no significant difference between conventional hybrids and LW pigs, and the difference between conventional hybrids and LIW pigs also decreased. Although the slightly lower fatness of LW pigs than that of LIW pigs was insignificant, conventional hybrids demonstrated considerably lower (*p* < 0.001) dorsal fat thickness compared with both Lithuanian breeds. The underbelly thickness of hybrids was also lower (*p* < 0.01 and *p* < 0.001, respectively) compared with LW and LIW pigs. LW pigs showed a higher (*p* < 0.05) loin area compared with LIW pigs; however, hybrid pigs demonstrated 11.89 cm^2^ and even 15.29 cm^2^ higher (*p* < 0.001) loin area than LW and LIW pig breeds, respectively.

Higher fatness and lower lean content were also found in the carcasses of other local pig breeds compared with modern breeds and their hybrids [20,21,22,23,24,25,49]. Females showed higher (*p* < 0.01) carcass length and lower (*p* < 0.05) dorsal fat thickness, except fat at the lumbar area, compared with castrated males. Higher subcutaneous fat thickness of castrated males is in agreement with the findings of other authors [50,51].

The chilled left side carcass of LIW pigs had lower (*p* < 0.05) weight of shoulder and middle part (belly) compared with LW and conventional hybrid pigs; however, the proportional weights of carcass cuts were insignificant (Table 3).

### 3.3. Meat Quality

LW and LIW pigs had the ability to deposit lipids, showing what resulted not only in high subcutaneous fat but also in a higher (*p* < 0.05 and *p* < 0.01, respectively) content of intramuscular fat (IMF) compared with lean conventional hybrids (Table 4). However, both Lithuanian local pig breeds demonstrating higher fatness at the same time showed a lower (*p* < 0.001 and *p* < 0.05, respectively) content of cholesterol compared with lean conventional hybrids.

Cholesterol contents determined for our local breeds were similar to those reported for Celta pigs [52]. A higher cholesterol content was found for the Pulawska breed [53]; however, the cholesterol content in this local Polish breed was also lower compared to Landrace. As the meat of suckling pigs had higher mean cholesterol levels compared to the meat of adult pigs [54], the higher slaughter age of local breeds could perhaps explain the lower cholesterol contents in their meat compared to modern breeds. However, other authors have reported discrepant results. The representative of lean breeds, Landrace, had less cholesterol compared to Mangalitsa pigs [55] and Chinese Huai pigs [56]. Large White also showed a lower content of cholesterol than Zlotnicka spotted, Pulawska and Mangalitsa pigs [10]. Although cholesterol can be derived from dietary sources and synthesised by the liver, different cholesterol biosyntheses can also be expressed by the pig genotype [56].

The ultimate pH of lean hybrid pig meat was lower (*p* < 0.05 and *p* < 0.001, respectively) compared with the meat of fatty LW and LIW pigs (Table 5). Higher pH in the meat of local pig breeds compared with commercial breeds was also detected by other authors [15,20,24,57]. Lightness (L*) of hybrid meat was higher (*p* < 0.001) than that of both local pig breeds, and these results are also consistent with the findings for other different local pigs [6,15,20,24]. Low pH and high L* values of hybrid pig muscles can indicate that conventional hybrids are more prone to meat defects. Hybrid pigs demonstrated lower (*p* < 0.001) meat redness (a*) than LW pigs and higher (*p* < 0.05) yellowness (b*) than LIW pigs. Higher redness compared to commercial breeds was also found in Pulawska [6,24], Basque [20] and Chinese pigs [15]. The highest (*p* < 0.01) saturation of meat colour (chroma) was found in the meat of LW pigs.

In agreement with the studies on other different breeds [6,15,20,24,57], conventional hybrids in the present study showed a higher (*p* < 0.05) drip loss compared with LW pigs and a higher (*p* < 0.01 and *p* < 0.001, respectively) cooking loss compared with LW and LIW pigs.

### 3.4. Meat Texture

Although it was indicated [58] that instrumental measurements could explain only a small portion (less than 20%) of the variability in sensory evaluation, both Warner–Bratzler shear force and texture profile analysis had a significant relationship with the sensory tenderness variables. In the present study, the Warner–Bratzler test demonstrated higher (*p* < 0.001) shear force of hybrid pigs compared with both Lithuanian pig breeds (Table 6). However, according to this measurement, Lithuanian breeds differed from each other. The meat of LW had higher (*p* < 0.001 and *p* < 0.05, respectively) shear force and toughness compared with LIW pigs.

There was a significant interaction (*p* < 0.01) concerning shear force between the pig genotype and gender. Hybrid and LW castrates showed higher shear force (1.59 N/m and 1.56 N/m, respectively) than hybrid gilts (1.44 N/m and 1.27 N/m, respectively), but the castrates of the LIW breed demonstrated lower shear force (1.01 N/m) than the gilts (1.04 N/m). Lower Warner–Bratzler shear force was indicated in the muscles of local Pulawska [24], Chinese [15] and Basque [20] pigs. However, another local breed, Celta, showed higher shear force than its crossbreds with Duroc and Landrace [25]. Warner–Bratzler shear force also indicated that raw meat was tougher in Cinta Senese than in Large White, but the differences disappeared after cooking [49]. In the present study, the longissimus muscle of lean conventional hybrids also demonstrated higher (*p* < 0.001) hardness by texture profile analysis (TPA) than the same muscles from both local pig breeds (Table 7). These results are consistent with the findings for the Polish Pulawska breed [24].

The muscles of females showed higher (*p* < 0.01) hardness compared with castrated males. Hybrid pigs also showed slightly higher (*p* < 0.05) springiness compared with LIW pigs, and there was an interaction (*p* < 0.01) for springiness between the genetic group and gender. The castrated males of the LIW breed showed lower springiness (0.828) than females (0.844), whereas the castrates of the LW breed and hybrids demonstrated the opposite results (0.845 and 0.847 for castrates and 0.840 and 0.844 for gilts, respectively).

### 3.5. Sensory Evaluation

During the blind test, the cooked loins from LW pigs demonstrated the most acceptable aroma for consumers; however, a significant (*p* < 0.05) difference was estimated only when compared to hybrids (Table 8). The loins from the LIW pig breed were found to be more juicy (*p* < 0.01 and *p* < 0.001, respectively) than the loins from LW and hybrid pigs. LW pigs also demonstrated higher (*p* < 0.001) juiciness of loins compared with lean conventional hybrids.

Lithuanian local breeds were also superior (*p* < 0.01 and *p* < 0.001) to hybrids in taste of loins and were scored higher in overall acceptability. After reviewing consumer attitudes toward pork, it was indicated that consumer liking increased as intramuscular fat levels and pH increased and WBSF decreased [59]. Similar differences in these parameters of loins between Lithuanian local pig breeds and conventional hybrids were also determined in the present study. Even trained taste panelists scored meat from local breeds (Preto Alentejano) as being juicier and having a richer taste than commercial meat [60].

As reported in other studies [21,61,62], the animal breed is an important factor influencing the consumer’s sensory perception. Consequently, a blind test provides an opportunity to avoid the influence of local consumers on the evaluation impartiality of national breeds.

No pronounced differences were found in aroma and taste between baked bellies from different pig genotypes. The bellies from LW and conventional hybrids were judged to be more juicy (*p* < 0.01) compared with the bellies from more fatty LIW pigs. A higher (*p* < 0.05) overall acceptability was observed for the lean bellies of conventional hybrids. It can be considered that such assessment is consistent with the consumer assessment of belly thickness because it is likely that consumers discriminate against products of excessive fatness [63]. Pork bellies are highly important and preferable primal cuts by consumers, the consumption of which has been rising in different countries [64,65,66]. However, the fact that one of the most important meat choice criteria for consumers is lean meat [67,68] apparently could have influenced such a rating of bellies that was the opposite of the loin rating. Hopes regarding the use of fatty bellies from local pig breeds can only be given by the fact that in our previous study [69], smoked backfat was recognisable by Lithuanian consumers almost as often as sausages, and this can suggest that certain numbers of even more fatty bellies may also have a niche for their use.

### 3.6. Fatty Acid Profiles

Pig genotype demonstrated an effect on the fatty acid profiles in LD (Table 9).

Conventional hybrids had a lower (*p* < 0.01) proportion of total saturated fatty acids (SFA), including individual C10:0, C12:0, C14:0 and C16:0 fatty acids compared with Lithuanian breeds. Hybrids had relatively 21.9% and 22.36% lower (*p* < 0.001) proportions of monounsaturated fatty acids (MUFA) than LIW and LW pigs, respectively. Lower proportions of SFA and MUFA in the muscle of commercial hybrids compared with indigenous Prestice Black-Pied pigs were reported by Nevrkla et al. [70]. Among the MUFA, only the concentration of C16:1n-9 fatty acid was higher (*p* < 0.001) in the LD of hybrids. As shown in our previous study [29], the differences in the fatty acid profiles of LD muscle between Lithuanian breeds were minimal. In the present study, a significant (*p* < 0.05) difference between Lithuanian breeds was found only for C20:1n-9 fatty acid. Conventional hybrids also had 2.36–2.45 times higher (*p* < 0.001) proportions of total polyunsaturated fatty acids (PUFA), including almost all individual polyunsaturated fatty acids, than Lithuanian local breeds.

Similar differences were found in the fatty acid profile of backfat (Table 10). The backfat of Lithuanian local pigs had 8.6–9.8% and 12.4–13.4% relatively higher (*p* < 0.001) proportions of SFA and MUFA and about 2.1 times lower proportions of PUFA compared with hybrids.

Although in our previous study [29], there were some differences found in the fatty acid profiles of LD muscle and backfat between the genders of Lithuanian breeds, and the gender effect on the fatty acid profile was also reported by other authors [70], in the present study, the gender did not affect the fatty acid profiles of either LD or backfat. No interactions between the pig genotype and gender for fatty acid profile were found.

The PUFA/SFA ratios (Table 11) in LD muscle and backfat of local breeds were below the minimum (0.4) recommended for the diet [71]. However, hybrids were in contrast to meet this recommendation.

Recently, nutritionists have focused on the balance in the diet between n-6 and n-3 of PUFA [71,72] and recommended this ratio be lower than 4. Although conventional hybrids demonstrated more favourable higher (*p* < 0.001) PUFA/SFA ratios both in LD muscle and backfat due to the greatest difference of the most abundant oleic fatty acid (C18:2n-6), local pigs showed more favourable lower (*p* < 0.001) n-6/n-3 PUFA ratios than hybrids. PUFA/SFA and *n*-6/*n*-3 PUFA ratios do not show the effects of MUFA; therefore, the lipid quality indices were calculated. All lipid quality indices were affected by the pig genotype. Lithuanian local pigs showed less favourable higher (*p* < 0.001 and *p* < 0.01, respectively) AI index in LD muscle and backfat compared with hybrids. Local pigs also demonstrated less favourable higher (*p* < 0.001) TI indices as well as lower (*p* < 0.001) h/H ratios. More favourable consumer health indices of commercial hybrids compared with local pigs were also reported by authors from other countries [70]. Lithuanian local pigs demonstrated more favourable lower (*p* < 0.001) PI indices in LD and subcutaneous lipids compared with conventional hybrids. Increases in fatty acid unsaturation may indicate the probability of oxidative rancidity [71,73]. In addition to the PI index, the quality of backfat is also shown by IV, which also represents a ratio of the number of unsaturated FA to saturated FA present in the adipose tissue and is very important in pork processing [73,74]. In this present study, the backfat of lean conventional hybrids demonstrated higher (*p* < 0.001) IV than local pigs. This finding is in agreement with the authors [46] who have reported that pork from pigs with greater adiposity has a lower IV. These authors have also reported about IV variation between the anatomical site and pig breed, suggesting that there may be genetic influences on IV.

## 4. Conclusions

Despite the higher fatness of local Lithuanian pig breeds compared with conventional hybrids, pigs of both local breeds demonstrated higher technological and eating meat quality, including lower cholesterol content but higher proportions of SFA and MUFA and lower proportion of PUFA than hybrids. The study demonstrated that during blind sensory evaluation, consumers preferred cooked loins from local breeds to those from hybrids, which had lower IMF, lower rate of pH and higher lightness and moisture losses. However, consumers preferred leaner baked bellies from conventional hybrids rather than more fatty bellies from local breeds, and this indicates that pork consumption patterns have been shifting towards lean meat. The peroxidisability index and iodine value in the lipids of local pig breeds showed a more favourable value for meat processing and storage compared with conventional hybrids. Fatty bellies from rare local breeds may be advisable for the production of smoked bellies. This information can be used to increase pork consumption choices and provides new insights for pork research into the promotion of local breeds.

## Figures and Tables

**Table 1:** Characteristics of slaughtered pigs from different genotypes.

Variables	Groups by Genotype			SED	Gender		SED	*p*-Value		
	LW *n =* 16	LIW *n* = 16	H *n* = 16		CM *n* = 24	F *n* = 24		Gr	G	Gr × G
Weight, kg	96.7	95.8	97.5	1.532	96.8	96.5	1.252	0.555	0.828	0.218
Age, days	202.3 ^e^	202.9 ^e^	125.8 ^f^	9.096	175.9	178.1	7.429	0.000	0.766	0.500
Daily gain, g	0.481 ^e^	0.475 ^e^	0.824 ^f^	0.044	0.597	0.589	0.036	0.000	0.825	0.882

LW—Lithuanian White; LIW—Lithuanian Indigenous Wattle; H—conventional hybrids; CM—males; F—females; Gr—group; G—gender; SED—standard error of difference; *p* values of GLM LSD tests for genotype groups were significantly different at ^e,f^ *p* < 0.001.

**Table 2:** Effects of pig genotype and gender on pig carcass characteristics.

Variables	Groups by Genotype			SED	Gender		SED	*p*-Value		
	LW *n* = 16	LIW *n* = 16	H *n* = 16		CM *n* = 24	F *n* = 24		Gr	G	Gr × G
Carcass weight, kg	67.4 ^a^	64.1 ^b^	68.4 ^a^	1.597	66.9	66.2	1.306	0.025	0.559	0.360
Dressing, %	69.6 ^a^	66.9 ^b^	70.5 ^a^	1.320	69.44	68.55	1.080	0.025	0.413	0.298
Carcass length, cm	96.6 ^c,e^	93.9 ^d,e^	99.9 ^f^	0.930	95.72 ^c^	97.90 ^d^	0.759	0.000	0.006	0.392
Bacon length, cm	83.11 ^c^	79.69 ^d^	82.76 ^c^	1.060	81.04	82.66	0.866	0.003	0.069	0.247
Backfat depth: at 1st rib, mm	50.63 ^e^	47.50 ^e^	32.48 ^f^	1.950	44.08	42.99	1.592	0.000	0.496	0.076
at 6/7th rib, mm	34.30 ^e^	35.00 ^e^	15.85 ^f^	1.961	30.48 ^a^	26.28 ^b^	1.601	0.000	0.012	0.189
at 10th rib, mm	30.78 ^e^	30.56 ^e^	14.76 ^f^	1.719	26.84 ^a^	23.89 ^b^	1.404	0.000	0.041	0.277
at last rib, mm	28.29 ^e^	28.63 ^e^	16.38 ^f^	1.650	26.07 ^a^	22.80 ^b^	1.348	0.000	0.020	0.326
at lumbar area, mm	30.07 ^e^	32.73 ^e^	13.40 ^f^	2.067	27.01	23.79	1.688	0.000	0.063	0.584
Depth of underbelly, mm	21.74 ^c^	23.44 ^e^	18.05 ^d,f^	1.118	20.52	21.64	0.913	0.000	0.226	0.501
Loin area, cm^2^	30.83 ^a,e^	27.43 ^b,e^	42.72 ^f^	1.638	33.26	34.05	1.338	0.000	0.559	0.240

LW—Lithuanian White; LIW—Lithuanian Indigenous Wattle; H—conventional hybrids; CM—castrated males; F—females; Gr—group; G—gender; SED—standard error of difference *p* values of GLM LSD tests for genotype and gender groups were significantly different at ^a,b^ *p* < 0.05; ^c,d^ *p* < 0.01; ^e,f^ *p* < 0.001.

**Table 3:** Effects of breed and gender on primal cuts of pig carcasses.

Variables	Groups by Genotype			SED	Gender		SED	*p*-Value		
	LW *n* = 16	LIW *n* = 16	H *n* = 16		CM *n* = 24	F *n* = 24		Gr	G	Gr × G
Weight of half carcass, kg	33.1 ^a^	30.9 ^b,c^	33.6 ^d^	0.858	32.7	32.4	0.700	0.008	0.715	0.512
Ham, kg	10.7	10.2	10.7	0.369	10.6	10.5	0.301	0.291	0.579	0.802
Ham, %	32.39	32.99	31.43	0.617	32.48	32.06	0.505	0.066	0.411	0.375
Shoulder, kg	11.5 ^a^	10.6 ^b^	11.7 ^a^	0.399	11.2	11.2	0.326	0.022	0.933	0.586
Shoulder, %	34.70	34.18	34.63	0.842	34.28	34.73	0.668	0.784	0.519	0.647
Middle part, kg	10.9 ^a^	10.2 ^b,c^	11.3 ^d^	0.321	10.8	10.7	0.262	0.005	0.815	0.086
Middle part, %	32.92	32.83	33.94	0.722	33.24	33.21	0.962	0.293	0.962	0.088

LW—Lithuanian White; LIW—Lithuanian Indigenous Wattle; H—conventional hybrids; CM—males; F—females; Gr—group; G—gender; SED—standard error of difference; *p* values of GLM LSD tests for genotype groups were significantly different at ^a,b^ *p* < 0.05 and ^c,d^ *p* < 0.01.

**Table 4:** Effects of pig genotype and gender on the chemical composition of pig LD muscles.

Variables	Groups by Genotype			SED	Gender		SED	*p*-Value		
	LW *n* = 16	LIW *n* = 16	H *n* = 16		CM *n* = 24	F *n* = 24		Gr	G	Gr × G
Dry matter, %	26.77	25.91	25.32	1.022	26.49	25.51	0.835	0.370	0.246	0.304
Protein, %	23.10	21.24	22.52	1.116	22.61	21.97	0.911	0.233	0.488	0.421
Fat, %	2.55 ^a^	2.90 ^c^	1.72 ^b,d^	0.384	2.59	2.19	0.314	0.014	0.205	0.845
Ash, %	0.92	0.87	0.95	0.039	0.93	0.90	0.032	0.106	0.409	0.632
Cholesterol, mg/100 g	36.44 ^e^	39.65 ^a^	44.24 ^f,b^	1.696	38.75	41.46	1.385	0.000	0.057	0.593

LW—Lithuanian White; LIW—Lithuanian Indigenous Wattle; H—conventional hybrids; CM—castrated males; F—females; Gr—group; G—gender; SED—standard error of difference; *p* values of GLM LSD tests for genotype and gender groups were significantly different at ^a,b^ *p* < 0.05; ^c,d^ *p* < 0.01; ^e,f^ *p* < 0.001.

**Table 5:** Effects of pig genotype and gender on LD muscles quality parameters.

Variables	Groups by Genotype			SED	Gender		SED	*p*-Value		
	LW *n* = 16	LIW *n* = 16	H *n* = 16		CM *n* = 24	F *n* = 24		Gr	G	Gr × G
pH	5.34 ^a^	5.45 ^e^	5.27 ^b,f^	0.049	5.36	5.34	0.040	0.004	0.488	0.092
Colour: L*	51.87 ^e^	53.10 ^e^	58.01 ^f^	1.040	54.31	54.35	0.849	0.000	0.964	0.075
a*	16.13 ^c,e^	15.13 ^d^	14.53 ^f^	0.352	15.23	15.31	0.288	0.000	0.792	0.490
b*	7.05 ^a^	6.01 ^b^	7.14 ^a^	0.433	6.77	6.69	0.354	0.022	0.803	0.192
C	17.64 ^c^	16.31 ^d^	16.21 ^d^	0.442	16.69	16.75	0.361	0.003	0.875	0.513
h	23.42 ^a^	21.51 ^e^	26.11 ^b,f^	1.163	23.89	23.47	0.950	0.002	0.666	0.114
Drip loss, %	5.23 ^a^	5.35	7.20 ^b^	0.909	5.67	6.18	0.742	0.071	0.493	0.280
Thawing loss, %	4.14	5.09	4.47	1.184	4.32	4.82	0.967	0.712	0.606	0.627
Cooking loss, %	33.83 ^c^	32.86 ^e^	37.85 ^d,f^	1.220	34.08	35.62	0.996	0.001	0.129	0.865

LW—Lithuanian White; LIW—Lithuanian Indigenous Wattle; H—conventional hybrids; CM—castrated males; F—females; Gr—group; G—gender; SED—standard error of difference; L*—lightness; a*—redness; b*—yellowness; C—chroma; h—hue angle; *p* values of GLM LSD tests for genotype groups were significantly different at ^a,b^ *p* < 0.05; ^c,d^ *p* < 0.01; ^e,f^ *p* < 0.001.

**Table 6:** Effects of pig genotype and gender on Warner–Bratzler test parameters of cooked LD muscles.

Variables	Groups by Genotype			SED	Gender		SED	*p*-Value		
	LW *n* = 16	LIW *n* = 16	H *n* = 16		CM *n* = 24	F *n* = 24		Gr	G	Gr × G
Shear force, N/m	1.21 ^e^	1.03 ^f,e′^	1.51 ^f,f′^	0.047	1.26	1.25	0.039	0.000	0.956	0.010
Toughness, N	99.42 ^a^	72.56 ^b^	96.81	12.975	83.41	96.76	10.218	0.079	0.162	0.065

LW—Lithuanian White; LIW—Lithuanian Indigenous Wattle; H—conventional hybrids; CM—castrated males; F—females; Gr—group; G—gender; SED—standard error of difference; *p* values of GLM LSD tests for genotype groups were significantly different at ^a,b^ *p* < 0.05; ^e,f and e′,f′^ *p* < 0.001.

**Table 7:** Effects of pig genotype and gender on cooked LD muscle parameters obtained by texture profile analysis.

Variables	Groups by Genotype			SED	Gender		SED	*p*-Value		
	LW *n* = 16	LIW *n* = 16	H *n* = 16		CM *n* = 24	F *n* = 24		Gr	G	Gr × G
Cohesiveness	2.11	2.10	2.20	0.061	2.14	2.14	0.050	0.217	0.995	0.386
Gumminess, N	18.03	19.15	21.94	2.494	19.09	20.32	2.031	0.265	0.545	0.188
Hardness, N	37.35 ^e^	33.74 ^e^	48.21 ^f^	2.997	36.32 ^c^	43.21 ^d^	2.447	0.000	0.005	0.310
Springiness	0.84	0.84 ^a^	0.85 ^b^	0.004	0.84	0.84	0.003	0.027	0.354	0.008
Chewiness, N	15.23	15.83	18.54	1.949	15.92	17.16	1.591	0.194	0.437	0.222

LW—Lithuanian White; LIW—Lithuanian Indigenous Wattle; H—conventional hybrids; CM—castrated males; F—females; Gr—group; G—gender; SED—standard error of difference; *p* values of GLM LSD tests for genotype and gender groups were significantly different at ^a,b^ *p* < 0.05; ^c,d^ *p* < 0.01 and ^e,f^ *p* < 0.001.

**Table 8:** Blind consumer evaluation of cooked loins and baked bellies from two local Lithuanian breeds and conventional hybrids.

Groups by Genotype	Aroma	Juiciness	Flavour/Taste	Overall Liking
Loin				
Lithuanian Indigenous Wattle	6.14 ± 0.12	6.45 ^c,e^ ± 0.12	6.44 ^e^ ± 0.11	6.36 ^c^ ± 0.11
Lithuanian White	6.28 ^a^ ± 0.10	6.06 ^d,e^ ± 0.13	6.36 ^c^ ± 0.11	6.23 ^a^ ± 0.11
Conventional hybrids	6.01 ^b^ ± 0.12	5.55 ^f^ ± 0.14	5.99 ^f,d^ ± 0.12	5.98 ^d,b^ ± 0.12
Belly				
Lithuanian Indigenous Wattle	6.14 ± 0.12	5.12 ^c^ ± 0.21	5.93 ± 0.17	5.81 ^a^ ± 0.18
Lithuanian White	6.06 ± 0.13	5.58 ^d^ ± 0.18	6.00 ± 0.15	5.94 ^a^ ± 0.15
Conventional hybrids	6.28 ± 0.12	5.85 ^d^ ± 0.15	6.17 ± 0.12	6.25 ^b^ ± 0.12

*p* values of GLM LSD tests for genotype groups were significantly different at ^a,b^ *p* < 0.05; ^c,d^ *p* < 0.01; ^e,f^ *p* < 0.001.

**Table 9:** Effects of pig genotype and gender on the fatty acid profile of Longissimus muscle.

Fatty Acids	Groups by Genotype			SED	Gender		SED	*p*-Value		
	LW *n* = 16	LIW *n* = 16	H *n* = 16		CM *n* = 24	F *n* = 24		Gr	G	Gr × G
C10:0	0.07 ^c^	0.08 ^e^	0.06 ^d,f^	0.006	0.07	0.07	0.005	0.001	0.071	0.964
C12:0	0.07 ^c^	0.07 ^c^	0.05 ^d^	0.004	0.06	0.06	0.004	0.007	0.256	0.207
C14:0	1.10 ^e^	1.16 ^e^	0.90 ^f^	0.046	1.08	1.03	0.038	0.000	0.158	0.404
C15:0	0.04 ^c^	0.04 ^c^	0.06 ^d^	0.007	0.04	0.05	0.006	0.007	0.260	0.738
C16:0	24.01 ^e^	24.00 ^e^	21.90 ^f^	0.430	23.61	23.00	0.351	0.000	0.089	0.321
C17:0	0.39	0.46	0.43	0.105	0.35	0.51	0.085	0.782	0.069	0.236
C18:0	10.92	10.88	11.09	0.370	10.94	10.98	0.302	0.837	0.898	0.765
C20:0	0.15	0.14	0.15	0.001	0.14	0.15	0.008	0.668	0.062	0.444
C22:0	0.11	0.11	0.12	0.013	0.10	0.12	0.011	0.786	0.113	0.493
SFA	36.84 ^c^	36.94 ^c^	34.74 ^d^	0.714	36.39	35.95	0.583	0.006	0.455	0.460
C14:1n-7	0.02	0.01	0.01	0.006	0.01	0.01	0.005	0.122	0.922	0.020
C16:1n7t	0.01	0.02	0.02	0.006	0.02	0.02	0.005	0.760	0.595	0.356
C16:1n-9	0.26 ^e^	0.28 ^c^	0.33 ^f,d^	0.016	0.28	0.29	0.013	0.000	0.358	0.967
C16:1n-7	3.30 ^e^	3.42 ^e^	2.25 ^f^	0.171	3.09	2.89	0.139	0.000	0.162	0.438
C17:1n-9	0.15	0.19	0.14	0.036	0.15	0.18	0.029	0.303	0.344	0.202
C18:1n-9t	0.24 ^a^	0.24 ^a^	0.19 ^b^	0.020	0.21	0.23	0.016	0.034	0.227	0.166
C18:1n-9	44.21 ^e^	43.38 ^e^	34.48 ^f^	1.041	41.02	40.36	0.851	0.000	0.442	0.711
C18:1n-7	4.27 ^e^	4.49 ^e^	3.30 ^f^	0.139	4.01	4.03	0.114	0.000	0.826	0.952
C20:1n-9	0.78 ^a,e^	0.88 ^b,e^	0.62 ^f^	0.039	0.75	0.76	0.032	0.000	0.742	0.168
C22:1n-9	0.04	0.05	0.02	0.014	0.03	0.04	0.011	0.159	0.166	0.396
MUFA	53.26 ^e^	52.95 ^e^	41.35 ^f^	1.172	49.56	48.81	0.958	0.000	0.438	0.676
C18:2n-6t	0.08 ^c^	0.07 ^c^	0.12 ^d^	0.013	0.09	0.09	0.010	0.001	0.586	0.402
C18:2n-6c,t	0.01	0.02	0.01	0.006	0.01	0.01	0.005	0.589	0.886	0.695
C18:2n-6t,c	0.03 ^c^	0.02	0.004 ^d^	0.009	0.02	0.02	0.007	0.007	0.278	0.423
C18:2n-6	5.59 ^e^	5.81 ^e^	13.94 ^f^	0.781	8.11	8.78	0.638	0.000	0.297	0.389
C18:3n-6	0.06 ^e^	0.06 ^e^	0.12 ^f^	0.013	0.07	0.09	0.011	0.000	0.214	0.875
C18:3n-3	0.41	0.46	0.47	0.030	0.43	0.46	0.024	0.062	0.250	0.582
C20:2n-6	0.17 ^e^	0.20 ^e^	0.39 ^f^	0.019	0.25	0.26	0.015	0.000	0.776	0.238
C20:3n-6	0.16 ^e^	0.18 ^e^	0.38 ^f^	0.034	0.23	0.25	0.027	0.000	0.367	0.344
C20:3n-3	0.09	0.09	0.10	0.010	0.09	0.10	0.008	0.400	0.203	0.448
C20:4n-6	1.07 ^e^	1.15 ^e^	3.30 ^f^	0.342	1.78	1.90	0.277	0.000	0.667	0.426
C20:5n-3	0.09 ^e^	0.09 ^e^	0.17 ^f^	0.019	0.10	0.13	0.015	0.000	0.156	0.498
C22:2n-6	0.01	0.00	0.02	0.010	0.01	0.02	0.008	0.153	0.297	0.451
C22:4n-6	0.18 ^e^	0.17 ^e^	0.45 ^f^	0.051	0.26	0.28	0.042	0.000	0.662	0.947
C22:5n-3	0.21 ^e^	0.21 ^e^	0.51 ^f^	0.047	0.28	0.33	0.038	0.000	0.169	0.608
C22:6n-3	0.09 ^e^	0.04 ^e^	0.27 ^f^	0.033	0.12	0.14	0.027	0.000	0.477	0.353
PUFA	8.24 ^e^	8.56 ^e^	20.25 ^f^	1.283	11.85	12.85	1.048	0.000	0.342	0.477
TFA	0.34	0.31	0.32	0.027	0.32	0.33	0.022	0.416	0.436	0.688
UFA	1.66 ^e^	1.55 ^e^	3.66 ^f^	0.409	2.20	2.38	0.333	0.000	0.590	0.508

LW—Lithuanian White; LIW—Lithuanian Indigenous Wattle; H—conventional hybrids; CM—castrated males; F—females; Gr—group; G—gender; SFAs—all identified saturated fatty acids; MUFAs—identified monounsaturated fatty acids; PUFAs—all identified polyunsaturated fatty acids; TFAs—all identified trans fatty acids; UFAs—unidentified fatty acids; SED—standard error of difference; *p* values of GLM LSD tests for genotype and gender groups were significantly different at ^a,b^ *p* < 0.05; ^c,d^ *p* < 0.01 and ^e,f^ *p* < 0.001.

**Table 10:** Effects of pig genotype and gender on the fatty acid profile of backfat.

Fatty Acids	Groups by Genotype			SED	Gender		SED	*p*-Value		
	LW *n* = 16	LIW *n* = 16	H *n* = 16		CM *n* = 24	F *n* = 24		Gr	G	Gr × G
C10:0	0.05	0.05	0.05	0.003	0.05	0.05	0.002	0.719	0.905	0.265
C12:0	0.06	0.06	0.06	0.003	0.06	0.06	0.002	0.809	0.613	0.239
C14:0	1.14	1.14	1.09	0.035	1.12	1.12	0.028	0.230	0.997	0.374
C15:0	0.04 ^c^	0.04 ^c^	0.05 ^d^	0.004	0.04	0.04	0.004	0.010	0.232	0.760
C16:0	24.60 ^c^	24.79 ^c^	22.85 ^d^	0.520	24.29	23.87	0.425	0.001	0.327	0.115
C17:0	0.28	0.25 ^c^	0.31 ^d^	0.022	0.27	0.29	0.018	0.025	0.249	0.581
C18:0	14.01 ^c^	13.44 ^a^	12.15 ^d,b^	0.518	13.24	13.16	0.423	0.003	0.855	0.426
C20:0	0.22 ^c^	0.21 ^c^	0.25 ^d^	0.012	0.22	0.23	0.010	0.005	0.620	0.109
C22:0	0.03	0.04	0.03	0.005	0.03	0.03	0.004	0.370	0.836	0.527
SFA	40.42 ^e^	40.01 ^c^	36.83 ^f,d^	0.934	39.32	38.85	0.763	0.001	0.540	0.151
C14:1n-7	0.01	0.01	0.02	0.005	0.01	0.01	0.004	0.130	0.240	0.268
C16:1n7t	0.03	0.03	0.03	0.003	0.03	0.03	0.003	0.655	0.222	0.909
C16:1n-9	0.33 ^e^	0.33 ^e^	0.43 ^f^	0.028	0.35	0.38	0.023	0.001	0.212	0.984
C16:1n-7	1.93 ^a^	2.20 ^b^	1.92 ^a^	0.115	2.00	2.03	0.094	0.033	0.827	0.839
C17:1n-9	0.22	0.22	0.21	0.028	0.21	0.22	0.022	0.922	0.468	0.218
C18:1n-9t	0.30 ^e^	0.27 ^e^	0.14 ^f^	0.025	0.23	0.25	0.020	0.000	0.257	0.608
C18:1n-9	43.87 ^e^	43.72 ^e^	39.04 ^f^	0.556	42.31	42.10	0.454	0.000	0.642	0.140
C18:1n-7	2.91 ^a,e^	3.13 ^b,e^	2.42 ^f^	0.107	2.76	2.88	0.088	0.000	0.172	0.595
C20:1n-9	1.07 ^a,e^	1.19 ^b,e^	0.86 ^f^	0.051	1.04	1.04	0.041	0.000	0.995	0.799
C22:1n-9	0.03	0.04 ^c^	0.03 ^d^	0.003	0.03	0.03	0.003	0.025	0.455	0.426
MUFA	50.69 ^e^	51.12 ^e^	45.09 ^f^	0.658	48.96	48.97	0.538	0.000	0.990	0.183
C18:2n-6t	0.05 ^c^	0.03 ^d^	0.05 ^c^	0.007	0.04	0.05	0.005	0.002	0.099	0.717
C18:2n-6c,t	0.03	0.03	0.03	0.005	0.03	0.03	0.004	0.193	0.563	0.067
C18:2n-6t,c	0.04 ^e^	0.05 ^e^	0.02 ^f^	0.007	0.03	0.04	0.005	0.000	0.401	0.384
C18:2n-6	6.88 ^e^	6.90 ^e^	15.60 ^f^	0.668	9.61	9.97	0.546	0.000	0.511	0.618
C18:3n-6	0.03 ^c^	0.04 ^a^	0.05 ^d,b^	0.005	0.04	0.04	0.004	0.005	0.906	0.423
C18:3n-3	0.84	0.79	0.82	0.049	0.79	0.84	0.040	0.609	0.236	0.075
C20:2n-6	0.36 ^e^	0.40 ^e^	0.63 ^f^	0.026	0.46	0.47	0.021	0.000	0.620	0.128
C20:3n-6	0.05 ^e^	0.06 ^e^	0.10 ^f^	0.006	0.07	0.07	0.005	0.000	0.534	0.250
C20:3n-3	0.15 ^c^	0.17 ^e^	0.12 ^d,f^	0.010	0.14	0.15	0.008	0.000	0.184	0.064
C20:4n-6	0.13 ^e^	0.14 ^e^	0.23 ^f^	0.019	0.16	0.17	0.015	0.000	0.318	0.837
C20:5n-3	0.01	0.00	0.01	0.003	0.01	0.01	0.003	0.073	0.809	0.643
C22:2n-6	0.00 ^e^	0.00 ^e^	0.02 ^f^	0.003	0.01	0.01	0.003	0.000	0.656	0.867
C22:4n-6	0.05 ^e^	0.06 ^a^	0.08 ^f,d^	0.008	0.07	0.07	0.006	0.000	0.674	0.997
C22:5n-3	0.07 ^e^	0.08 ^c^	0.10 ^f,d^	0.007	0.08	0.08	0.006	0.001	0.632	0.744
C22:6n-3	0.01 ^e^	0.01 ^e^	0.05 ^f^	0.008	0.02	0.02	0.006	0.000	0.849	0.171
PUFA	8.69 ^e^	8.73 ^e^	17.89 ^f^	0.769	11.54	12.01	0.628	0.000	0.464	0.544
TFA	0.42 ^e^	0.38 ^e^	0.26 ^f^	0.031	0.34	0.37	0.025	0.000	0.215	0.983
UFA	0.20	0.14	0.19	0.029	0.18	0.18	0.024	0.121	0.976	0.064

LW—Lithuanian White; LIW—Lithuanian Indigenous Wattle; H—conventional hybrids; CM—castrated males; F—females; Gr—group; G—gender; SFAs—all identified saturated fatty acids; MUFAs—identified monounsaturated fatty acids; PUFAs—all identified polyunsaturated fatty acids; TFAs—all identified trans fatty acids; UFAs—unidentified fatty acids; SED—standard error of difference; *p* values of GLM LSD tests for genotype and gender groups were significantly different at ^a,b^ *p* < 0.05; ^c,d^ *p* < 0.01 and ^e,f^ *p* < 0.001.

**Table 11:** Fatty acid ratios and lipid quality indices in LD muscle and backfat.

Variables	Groups by Genotype			SED	Gender		SED	*p*-Value		
	LW *n* = 16	LIW *n* = 16	H *n* = 16		CM *n* = 24	F *n* = 24		Gr	G	Gr × G
LD muscle										
PUFA/SFA	0.23 ^e^	0.23 ^e^	0.60 ^f^	0.045	0.34	0.37	0.036	0.000	0.407	0.511
n-6/n-3 PUFA	8.25 ^e^	8.69 ^e^	12.35 ^f^	0.350	9.83	9.70	0.404	0.000	0.761	0.094
AI	0.46 ^e^	0.47 ^e^	0.42 ^f^	0.013	0.46	0.44	0.010	0.000	0.149	0.351
TI	1.09 ^c^	1.09 ^c^	0.98 ^d^	0.035	1.07	1.04	0.028	0.003	0.271	0.532
h/H	2.27 ^e^	2.25 ^e^	2.55 ^f^	0.065	2.31	2.39	0.053	0.000	0.138	0.332
PI	16.13 ^e^	16.39 ^e^	38.85 ^f^	2.782	22.80	24.78	2.271	0.000	0.386	0.574
Backfat										
PUFA/SFA	0.22 ^e^	0.22 ^e^	0.50 ^f^	0.028	0.31	0.32	0.023	0.000	0.668	0.410
n-6/n-3 PUFA	7.20 ^e^	7.40 ^e^	15.54 ^f^	0.404	9.98	10.11	0.330	0.000	0.717	0.695
AI	0.49 ^c^	0.49 ^c^	0.44 ^d^	0.017	0.48	0.47	0.014	0.002	0.385	0.129
TI	1.23 ^c^	1.21 ^c^	1.06 ^d^	0.050	1.18	1.15	0.041	0.003	0.445	0.169
h/H	2.16 ^e^	2.14 ^e^	2.48 ^f^	0.082	2.24	2.28	0.067	0.000	0.555	0.084
PI	12.01 ^e^	12.11 ^e^	21.90 ^f^	0.924	15.05	15.63	0.754	0.000	0.443	0.578
IV	57.93 ^e^	58.18 ^e^	68.18 ^f^	1.302	61.05	61.81	1.063	0.000	0.475	0.240

LW—Lithuanian White; LIW—Lithuanian Indigenous Wattle; H—conventional hybrids; CM—castrated males; F—females; Gr—group; G—gender; PUFAs/SFAs—ratio of PUFAs to SFAs; n-6/n-3—ratio of n-6 PUFAs to n-3 PUFAs, AI—atherogenic index, TI—thrombogenic index, h/H—hypocholesterolemic/hypercholesterolemic ratio, PI—peroxidisability index. IV—iodine value; SED—standard error of difference; *p* values of GLM LSD tests for genotype and gender groups were significantly different at ^c,d^ *p* < 0.01 and ^e,f^ *p* < 0.001.

## Data Availability

The original contributions presented in the study are included in the article, further inquiries can be directed to the corresponding author.
